# Hesitant Fuzzy Soft Subalgebras and Ideals in *BCK/BCI*-Algebras

**DOI:** 10.1155/2014/763929

**Published:** 2014-10-14

**Authors:** Young Bae Jun, Sun Shin Ahn, G. Muhiuddin

**Affiliations:** ^1^Department of Mathematics Education, Gyeongsang National University, Jinju 660-701, Republic of Korea; ^2^Department of Mathematics Education, Dongguk University, Seoul 100-715, Republic of Korea; ^3^Department of Mathematics, University of Tabuk, Tabuk 71491, Saudi Arabia

## Abstract

As a link between classical soft sets and hesitant fuzzy sets, the notion of hesitant fuzzy soft sets is introduced and applied to a decision making problem in the papers by Babitha and John (2013) and Wang et al. (2014). The aim of this paper is to apply hesitant fuzzy soft set for dealing with several kinds of theories in *BCK/BCI*-algebras. The notions of hesitant fuzzy soft subalgebras and (closed) hesitant fuzzy soft ideals are introduced, and related properties are investigated. Relations between a hesitant fuzzy soft subalgebra and a (closed) hesitant fuzzy soft ideal are discussed. Conditions for a hesitant fuzzy soft set to be a hesitant fuzzy soft subalgebra are given, and conditions for a hesitant fuzzy soft subalgebra to be a hesitant fuzzy soft ideal are provided. Characterizations of a (closed) hesitant fuzzy soft ideal are considered.

## 1. Introduction

Nowadays, even if Molodtsov's soft set theory (see [1]) is a newly emerging mathematical tool to handle uncertainty, the classical soft sets are not appropriate to deal with imprecise and fuzzy parameters. In order to overcome this situation, Maji et al. [2] introduced the concept of fuzzy soft sets as a generalization of the standard soft sets and presented an application of fuzzy soft sets in a decision making problem. The notions of Atanassov's intuitionistic fuzzy sets, type 2 fuzzy sets, fuzzy multisets, and so forth are a generalization of fuzzy sets. As another generalization of fuzzy sets, Torra [3] introduced the notion of hesitant fuzzy sets which are very useful to express peoples hesitancy in daily life. The hesitant fuzzy set is a very useful tool to deal with uncertainty, which can be accurately and perfectly described in terms of the opinions of decision makers. Xu and Xia [4] proposed a variety of distance measures for hesitant fuzzy sets, based on which the corresponding similarity measures can be obtained. They investigated the connections of the aforementioned distance measures and further develop a number of hesitant ordered weighted distance measures and hesitant ordered weighted similarity measures. Also, hesitant fuzzy set theory is used in decision making problem, and so forth (see [5–9]), and is applied to residuated lattices and* MTL*-algebras (see [10, 11]). In soft set theory membership is decided by adequate parameters, and hesitant fuzzy set employs all possible values for the membership of an element. The soft set model has been combined with other mathematical models, for example, fuzzy soft sets by combining fuzzy sets and soft sets (see [2]), intuitionistic fuzzy soft sets which are based on a combination of intuitionistic fuzzy sets and soft sets (see [12, 13]), interval-valued fuzzy soft sets (see [14]), trapezoidal fuzzy soft sets (see [15]), and forth. As a link between classical soft sets and hesitant fuzzy sets, the notion of hesitant fuzzy soft sets is introduced and applied to a decision making problem in [6, 16].

In this paper, we apply the notion of hesitant fuzzy soft sets to subalgebras and ideals in* BCK/BCI*-algebras. We introduce the notion of hesitant fuzzy soft subalgebras and (closed) hesitant fuzzy soft ideals and investigate related properties. We consider relations between a hesitant fuzzy soft subalgebra and a hesitant fuzzy soft ideal. We provide conditions for a hesitant fuzzy soft set to be a hesitant fuzzy soft subalgebra. We also give conditions for a hesitant fuzzy soft subalgebra to be a hesitant fuzzy soft ideal. We discuss characterizations of a (closed) hesitant fuzzy soft ideal.

## 2. Preliminaries

A* BCK/BCI*-algebra is an important class of logical algebras introduced by K. Iséki and was extensively investigated by several researchers.

An algebra (*X*; ∗, 0) of type (2,0) is called a* BCI-algebra* if it satisfies the following conditions:

(I)* * (∀*x*, *y*, *z* ∈ *X*)  (((*x*∗*y*)∗(*x*∗*z*))∗(*z*∗*y*) = 0),
(II)* * (∀*x*, *y* ∈ *X*)  ((*x*∗(*x*∗*y*))∗*y* = 0),
(III)* * (∀*x* ∈ *X*)  (*x*∗*x* = 0),
(IV)* * (∀*x*, *y* ∈ *X*)  (*x*∗*y* = 0, *y*∗*x* = 0⇒*x* = *y*).
 If a* BCI*-algebra *X* satisfies the following identity:
(V)* * (∀*x* ∈ *X*)  (0∗*x* = 0),
 then *X* is called a* BCK-algebra*; any* BCK*-algebra *X* satisfies the following axioms:
  (a1)* * (∀*x* ∈ *X*)  (*x*∗0 = *x*),
  (a2)* * (∀*x*, *y*, *z* ∈ *X*)  (*x* ≤ *y*⇒*x*∗*z* ≤ *y*∗*z*, *z*∗*y* ≤ *z*∗*x*),
  (a3)* * (∀*x*, *y*, *z* ∈ *X*)  ((*x*∗*y*)∗*z* = (*x*∗*z*)∗*y*),
  (a4)* * (∀*x*, *y*, *z* ∈ *X*)  ((*x*∗*z*)∗(*y*∗*z*) ≤ *x*∗*y*),
   where *x* ≤ *y* if and only if *x*∗*y* = 0;
   Any* BCI*-algebra *X* satisfies the following axioms:
  (a5)* * (∀*x*, *y*, *z* ∈ *X*)  (0∗(0∗((*x*∗*z*)∗(*y*∗*z*))) = (0∗*y*)∗(0∗*x*)).

(∀*x*, *y* ∈ *X*)  (0∗(0∗(*x*∗*y*)) = (0∗*y*)∗(0∗*x*)). 

A nonempty subset *S* of a* BCK/BCI*-algebra *X* is called a* subalgebra* of *X* if *x*∗*y* ∈ *S* for all *x*, *y* ∈ *S*.

A subset *I* of a* BCK/BCI*-algebra *X* is called an* ideal* of *X* if it satisfies

(1) $$\begin{array}{c} 0\in I,\\ \left(\forall x,y\in X\right)\;\left(x*y\in I,y\in I⟹x\in I\right). \end{array}$$

An ideal *I* of a* BCI*-algebra *X* is said to be* closed* if 0∗*x* ∈ *I* for every *x* ∈ *I*.

We refer the reader to the books [17, 18] for further information regarding* BCK/BCI*-algebras.

**Definition 1 (see [3]).**

A* hesitant fuzzy set* on a reference set (or an initial universe set) *U* is defined in terms of a function that when applied to *U* returns a subset of [0,1], which can be viewed as the following mathematical representation:

(2) $$\begin{array}{c} H≔\left\{\left(u,h_{H}\left(u\right)\right)\;|\;u\in U\right\}, \end{array}$$

where *h*_*H*_ : *U* → *P*([0,1]).

Denote by *HF*(*U*) the set of all hesitant fuzzy sets on a reference set (or an initial universe set) *U*.

**Definition 2 (see [19]).**

Let *X* be a* BCK/BCI*-algebra. A hesitant fuzzy set,

(3) $$\begin{array}{c} H≔\left\{\left(x,h_{H}\left(x\right)\right)\;|\;x\in X\right\}, \end{array}$$

on *X* is called a* hesitant fuzzy subalgebra* of *X* if it satisfies

(4) $$\begin{array}{c} \left(\forall x,y\in X\right)\;\left(h_{H}\left(x*y\right)⊇h_{H}\left(x\right)\cap h_{H}\left(y\right)\right). \end{array}$$

**Definition 3 (see [19]).**

Let *X* be a* BCK/BCI*-algebra. A hesitant fuzzy set,

(5) $$\begin{array}{c} H≔\left\{\left(x,h_{H}\left(x\right)\right)\;|\;x\in X\right\}, \end{array}$$

on *X* is called a* hesitant fuzzy ideal* of *X* if it satisfies

(6) $$\begin{array}{c} \left(\forall x,y\in X\right)\;\left(h_{H}\left(x*y\right)\cap h_{H}\left(y\right)\subseteq h_{H}\left(x\right)\subseteq h_{H}\left(0\right)\right). \end{array}$$

**Definition 4 (see [6, 16]).**

A pair $\left(\tilde{H},A\right)$ is called a* hesitant fuzzy soft set* over a reference set *U*, where $\tilde{H}$ is a mapping given by

(7) $$\begin{array}{c} \tilde{H}:A⟶HF\left(U\right). \end{array}$$

## 3. Hesitant Fuzzy Soft Subalgebras

In what follows let *E* be a set of parameters and we take a* BCK/BCI*-algebra *X* as a reference set unless otherwise specified.

**Definition 5 .**

For a subset *A* of *E*, a hesitant fuzzy soft set $\left(\tilde{H},A\right)$ over *X* is called a* hesitant fuzzy soft subalgebra* based on *e* ∈ *A* (briefly, *e-hesitant fuzzy soft subalgebra*) over *X* if the hesitant fuzzy set,

(8) $$\begin{array}{c} \tilde{H}\left[e\right]≔\left\{\left(x,h_{\tilde{H}[e]}\left(x\right)\right)\;|\;x\in X\right\}, \end{array}$$

on *X* is a hesitant fuzzy subalgebra of *X*. If $\left(\tilde{H},A\right)$ is an *e*-hesitant fuzzy soft subalgebra over *X* for all *e* ∈ *A*, we say that $\left(\tilde{H},A\right)$ is a* hesitant fuzzy soft subalgebra*.

**Example 6 .**

Let *X* = {0, *a*, *b*, *c*, *d*} be a* BCK*-algebra with the following Cayley table.

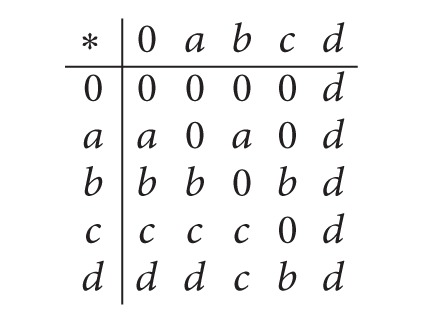
(9)
Consider a set of parameters *E*≔{*e*_1_, *e*_2_, *e*_3_, *e*_4_, *e*_5_}.

(1) Let $\left(\tilde{H},A\right)$ be a hesitant fuzzy soft set over *X* where *A* = {*e*_1_, *e*_2_, *e*_3_} which is given in Table 1.

It is routine to verify that $\tilde{H}[e_{1}]$, $\tilde{H}[e_{2}]$, and $\tilde{H}[e_{3}]$ are hesitant fuzzy subalgebras of *X*; that is, $\left(\tilde{H},A\right)$ is a hesitant fuzzy soft subalgebra over *X* based on parameters “*e*_1_,” “*e*_2_,” and “*e*_3_.” Therefore $\left(\tilde{H},A\right)$ is a hesitant fuzzy soft subalgebra over *X*.

(2) Let $\left(\tilde{G},B\right)$ be a hesitant fuzzy soft set over *X* where *B* = {*e*_3_, *e*_4_, *e*_5_} which is defined in Table 2.

It is easily checked that $\left(\tilde{G},B\right)$ is a hesitant fuzzy soft subalgebra based on parameters “*e*_3_” and “*e*_5_” over *X*. But it is not an *e*_4_-hesitant fuzzy soft subalgebra over *X* since the hesitant fuzzy set,

(10) $$\begin{array}{c} \tilde{G}\left[e_{4}\right]≔\left\{\left(x,h_{\tilde{G}[e_{4}]}\left(x\right)\right)\;|\;x\in X\right\}, \end{array}$$

is not a hesitant fuzzy subalgebra of *X*. In fact,

(11) hG~[e4](a∗c)=hG~[e4](0)={0.3}⊉[0.3,0.4]=hG~[e4](a)∩hG~[e4](c).

**Example 7 .**

Let *X* = {0,1, 2, *a*, *b*} be a* BCI*-algebra with the following Cayley table.

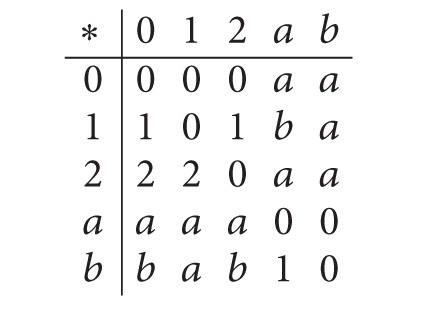
(12)
Given a set *E*≔{*e*_1_, *e*_2_, *e*_3_} of parameters, let $\left(\tilde{H},E\right)$ be a hesitant fuzzy soft set over *X* which is described in Table 3.

Then $\left(\tilde{H},E\right)$ is a hesitant fuzzy soft subalgebra over *X*.

**Proposition 8 .**

If $\left(\tilde{H},A\right)$ is a hesitant fuzzy soft subalgebra over *X*, then

(13) $$\begin{array}{c} \left(\forall x\in X\right)\;\left(h_{\tilde{H}[e]}\left(x\right)\subseteq h_{\tilde{H}[e]}\left(0\right)\right), \end{array}$$

where *e* is any parameter in *A*.

**Proof**

For any *x* ∈ *X* and *e* ∈ *A*, we have

(14) $$\begin{array}{c} h_{\tilde{H}[e]}\left(0\right)=h_{\tilde{H}[e]}\left(x*x\right)⊇h_{\tilde{H}[e]}\left(x\right)\cap h_{\tilde{H}[e]}\left(x\right)=h_{\tilde{H}[e]}\left(x\right). \end{array}$$

This completes the proof.

**Theorem 9 .**

Let $\left(\tilde{H},A\right)$ be a hesitant fuzzy soft subalgebra over *X*. If *B* is a subset of *A*, then $\left({\tilde{H}|}_{B},B\right)$ is a hesitant fuzzy soft subalgebra over *X*.

**Proof**

Straightforward.

The following example shows that there exists a hesitant fuzzy soft set $\left(\tilde{H},A\right)$ over *X* such that

- $\left(\tilde{H},A\right)$ is not a hesitant fuzzy soft subalgebra over *X*,
- there exists a subset *B* of *A* such that $\left({\tilde{H}|}_{B},B\right)$ is a hesitant fuzzy soft subalgebra over *X*.

**Example 10 .**

Let *X* = {0, *a*, *b*, *c*, *d*} be a* BCK*-algebra as in Example 6. Consider a set *A* = {*e*_*i*_∣*i* = 1,2, 3,4, 5} of parameters. Let $\left(\tilde{H},A\right)$ be a hesitant fuzzy soft set over *X* which is described in Table 4.

Then two hesitant fuzzy sets,

(15) $$\begin{array}{c} \tilde{H}\left[e_{4}\right]≔\left\{\left(x,h_{\tilde{H}[e_{4}]}\left(x\right)\right)\;|\;x\in X\right\},\\ \tilde{H}\left[e_{5}\right]≔\left\{\left(x,h_{\tilde{H}[e_{5}]}\left(x\right)\right)\;|\;x\in X\right\}, \end{array}$$

on *X* are not hesitant fuzzy subalgebras of *X* since

(16) hH~[e4](a∗c)=hH~[e4](0)={0,1}⊉[0.1,0.2]=hH~[e4](a)∩hH~[e4](c),hH~[e5](b∗b)=hH~[e5](0)=[0.1,0.2]⊉[0.1,0.5]=hH~[e5](b)∩hH~[e5](b),

respectively. Therefore $\left(\tilde{H},A\right)$ is not a hesitant fuzzy soft subalgebra over *X*. But if we take *B* = {*e*_1_, *e*_2_, *e*_3_}, then $\left({\tilde{H}|}_{B},B\right)$ is a hesitant fuzzy soft subalgebra over *X*.

## 4. Hesitant Fuzzy Soft Ideals

**Definition 11 .**

Let $\left(\tilde{H},A\right)$ be a hesitant fuzzy soft set over *X* where *A* is a subset of *E*. Given *e* ∈ *A*, $\left(\tilde{H},A\right)$ is called a* hesitant fuzzy soft ideal* based on *e* (briefly, *e-hesitant fuzzy soft ideal*) over *X* if the hesitant fuzzy set,

(17) $$\begin{array}{c} \tilde{H}\left[e\right]≔\left\{\left(x,h_{h_{\tilde{H}[e]}}\left(x\right)\right)\;|\;x\in X\right\}, \end{array}$$

on *X* is a hesitant fuzzy ideal of *X*. If $\left(\tilde{H},A\right)$ is an *e*-hesitant fuzzy soft ideal over *X* for all *e* ∈ *A*, we say that $\left(\tilde{H},A\right)$ is a* hesitant fuzzy soft ideal* over *X*.

**Example 12 .**

Let *X*≔{apple, banana, grape, melon, peach} be a reference set, and consider an operation ⊗ which produces the following products:

(18) $$\begin{array}{c} \text{apple}⊗x=\text{apple}\;\forall x\in X,\\ \text{banana}⊗y=\{\begin{array}{cc} \text{apple} & \text{if}\;y\in \left\{\text{banana},\text{peaqch},\text{melon}\right\},\\ \text{banana} & \text{if}\;y\in \left\{\text{apple},\text{grape}\right\}, \end{array}\\ \text{grape}⊗z=\{\begin{array}{cc} \text{grape} & \text{if}\;z\in \left\{\text{apple},\text{banana},\text{melon}\right\},\\ \text{apple} & \text{if}\;z\in \left\{\text{grape},\text{peach}\right\}, \end{array}\\ \text{peach}⊗u=\{\begin{array}{cc} \text{peach} & \text{if}\;u=\text{apple},\\ \text{banana} & \text{if}\;u=\text{grape},\\ \text{apple} & \text{if}\;u=\text{peach},\\ \text{grape} & \text{if}\;u\in \left\{\text{banana},\text{melon}\right\}, \end{array}\\ \text{melon}⊗v=\{\begin{array}{cc} \text{apple} & \text{if}\;v=\text{melon},\\ \text{melon} & \text{if}\;v\in \left\{\text{apple},\text{grape}\right\},\\ \text{banana} & \text{if}\;v\in \left\{\text{banana},\text{peach}\right\}. \end{array} \end{array}$$

Then (*X*, ⊗, apple) is a* BCK*-algebra. Consider a set of parameters

(19) $$\begin{array}{c} E≔\left\{\text{cat},\text{cow},\text{dog},\text{duck},\text{horse},\text{pig}\right\}. \end{array}$$

Let $\left(\tilde{H},E\right)$ be a hesitant fuzzy soft set over *X* which is described in Table 5.

Then $\left(\tilde{H},E\right)$ is a hesitant fuzzy soft ideal over *X* based on parameters “cat,” “cow,” “dog,” “duck,” and “pig.” But $\left(\tilde{H},E\right)$ is not a hesitant fuzzy soft ideal of *X* based on parameter “horse” since

(20) H~[horse](melon) ={0.3}⊉[0.3,0.4) =H~[horse](melon⊗peach)∩H~[horse](peach).

In general, we know that horses like grapes best of all. In the above example, we know that $\left(\tilde{H},E\right)$ is not a hesitant fuzzy soft ideal of *X* based on parameter “horse.” This means that if a horse likes a grape better than the others, then $\left(\tilde{H},E\right)$ cannot be a hesitant fuzzy ideal over *X*.

**Proposition 13 .**

Every hesitant fuzzy soft ideal $\left(\tilde{H},A\right)$ over *X* satisfies the following condition:

(21) (∀e∈A)  (∀x,y,z∈X)(x∗y≤z⟹hH~[e](x)⊇hH~[e](y)∩hH~[e](z)).

**Proof**

Let *e* ∈ *A* and *x*, *y*, *z* ∈ *X* be such that *x*∗*y* ≤ *z*. Then (*x*∗*y*)∗*z* = 0, and so

(22) hH~[e](x∗y)⊇hH~[e]((x∗y)∗z)∩hH~[e](z)=hH~[e](0)∩hH~[e](z)=hH~[e](z).

It follows that $h_{\tilde{H}[e]}(x)⊇h_{\tilde{H}[e]}(x*y)\cap h_{\tilde{H}[e]}(y)⊇h_{\tilde{H}[e]}(y)\cap h_{\tilde{H}[e]}(z)$. This completes the proof.

**Theorem 14 .**

Let $\left(\tilde{H},A\right)$ be a hesitant fuzzy soft set over *X* which satisfies the condition (13) and (21). Then $\left(\tilde{H},A\right)$ is a hesitant fuzzy soft ideal over *X*.

**Proof**

Let *e* ∈ *A*. Since *x*∗(*x*∗*y*) ≤ *y* for all *x*, *y* ∈ *X*, it follows from (21) that

(23) $$\begin{array}{c} h_{\tilde{H}[e]}\left(x\right)⊇h_{\tilde{H}[e]}\left(x*y\right)\cap h_{\tilde{H}[e]}\left(y\right). \end{array}$$

Hence $\left(\tilde{H},A\right)$ is an *e*-hesitant fuzzy soft ideal over *X*. Since *e* is arbitrary, we know that $\left(\tilde{H},A\right)$ is a hesitant fuzzy soft ideal over *X*.

**Theorem 15 .**

In a* BCK*-algebra *X*, every hesitant fuzzy soft ideal (based on a parameter) over *X* is a hesitant fuzzy soft subalgebra (based on the same parameter) over *X*.

**Proof**

For any *e* ∈ *A*, let $\left(\tilde{H},A\right)$ be an *e*-hesitant fuzzy soft ideal over *X*. Then

(24) hH~[e](x∗y)⊇hH~[e]((x∗y)∗x)∩hH~[e](x)=hH~[e]((x∗x)∗y)∩hH~[e](x)=hH~[e](0∗y)∩hH~[e](x)=hH~[e](0)∩hH~[e](x)⊇hH~[e](x)∩hH~[e](y),

for all *x*, *y* ∈ *X*, and so $\left(\tilde{H},A\right)$ is an *e*-hesitant fuzzy soft subalgebra over *X*.

The following example shows that the converse of Theorem 15 is not true in general.

**Example 16 .**

Let *X*≔{apple, banana, grape, melon, peach} be a universe, and consider an operation ⊟ which produces the following products:

(25) apple⊟x={appleif  x∈{grape,peach,melon},grapeif  x∈{apple,banana},banana⊟y ={bananaif  y∈{apple,grape,peach,melon},grapeif  x=banana,grape⊟z=grape ∀z∈U,melon⊟v={melonif  v∈{grape,peach},grapeif  v∈{apple,banana,melon},peach⊟u={peachif  u∈{grape,melon},grapeif  u∈{apple,peach,banana}.

Then (*X*, ⊟, grape) is a* BCK*-algebra. Consider a set of parameters:

(26) $$\begin{array}{c} B≔\left\{\text{duck},\text{horse},\text{pig}\right\}. \end{array}$$

Let $\left(\tilde{H},B\right)$ be a hesitant fuzzy soft set over *X* which is given in Table 6.

Then $\left(\tilde{H},B\right)$ is a hesitant fuzzy soft subalgebra over *X*, but it is not a hesitant fuzzy soft ideal over *X* based on parameter “horse” since

(27) H~[horse](peach) ={0.2}⊉[0.2,0.5) =H~[horse](peach⊗apple)∩H~[horse](apple).

We provide a condition for a hesitant fuzzy soft subalgebra to be a hesitant fuzzy soft ideal.

**Theorem 17 .**

Let $\left(\tilde{H},A\right)$ be a hesitant fuzzy soft subalgebra over *X*. Then $\left(\tilde{H},A\right)$ is a hesitant fuzzy soft ideal over *X* if and only if the condition (21) is valid.

**Proof**

Necessity is by Proposition 13.

Conversely, assume that the condition (21) is valid. Since *x*∗(*x*∗*y*) ≤ *y* for all *x*, *y* ∈ *X*, it follows that $h_{\tilde{H}[e]}(x)⊇h_{\tilde{H}[e]}(x*y)\cap h_{\tilde{H}[e]}(y)$ for all *e* ∈ *A* and *x*, *y* ∈ *X*. Combining this and (13), we know that $\left(\tilde{H},A\right)$ is a hesitant fuzzy soft ideal over *X*.

**Proposition 18 .**

Every hesitant fuzzy soft ideal $\left(\tilde{H},A\right)$ over a* BCI*-algebra *X* satisfies the following inequality:

(28) $$\begin{array}{c} \left(\forall e\in A\right)\left(\forall x\in X\right)\;\left(h_{\tilde{H}[e]}\left(0*\left(0*x\right)\right)⊇h_{\tilde{H}[e]}\left(x\right)\right). \end{array}$$

**Proof**

Let $\left(\tilde{H},A\right)$ be a hesitant fuzzy soft ideal of a* BCI*-algebra *X*. Then

(29) hH~[e](0∗(0∗x))⊇hH~[e]((0∗(0∗x))∗x)∩hH~[e](x)=hH~[e](0)∩hH~[e](x)=hH~[e](x),

for all *e* ∈ *A* and *x* ∈ *X*.

The following example shows that a hesitant fuzzy soft ideal over a* BCI*-algebra (based on a parameter) may not be a hesitant fuzzy soft subalgebra (based on the same parameter).

**Example 19 .**

Let *X* be a reference set which consists of all nonzero rational numbers. Let ÷ be a binary operation which is defined as division in general. Then (*X*, ÷, 1) is a* BCI*-algebra. For a subset *A* of *E*, let $\left(\tilde{H},A\right)$ be a hesitant fuzzy soft set over *X* in which $h_{\tilde{H}[e]}$ is defined as follows:

(30) $$\begin{array}{c} h_{\tilde{H}[e]}\left(x\right)≔\{\begin{array}{cc} \left[0.2,0.7\right] & \text{if}\;x\in Z^{*},\\ \left[0.5,0.7\right] & \text{otherwise}, \end{array} \end{array}$$

for all *e* ∈ *A* and *x* ∈ *X*, where *Z* ^*^ is the set of all nonzero integers. Then $\left(\tilde{H},A\right)$ is a hesitant fuzzy soft ideal over *X*, but it is not a hesitant fuzzy soft subalgebra over *X* since

(31) hH~[e](3÷2)=[0.5,0.7]⊉[0.2,0.7]=hH~[e](3)∩hH~[e](2).

**Definition 20 .**

A hesitant fuzzy ideal *H*≔{(*x*, *h*_*H*_(*x*))∣*x* ∈ *X*} of a *BCI*-algebra *X* is said to be* closed* if *h*_*H*_(*x*)⊆*h*_*H*_(0∗*x*) for all *x* ∈ *X*.

**Definition 21 .**

A hesitant fuzzy soft ideal $\left(\tilde{H},A\right)$ over a *BCI*-algebra *X* based on a parameter *e* ∈ *A* is said to be* closed* if the hesitant fuzzy set,

(32) $$\begin{array}{c} \tilde{H}\left[e\right]≔\left\{\left(x,h_{\tilde{H}[e]}\left(x\right)\right)\;|\;x\in X\right\}, \end{array}$$

on *X* is a closed hesitant fuzzy ideal of *X*.

**Example 22 .**

The hesitant fuzzy soft subalgebra $\left(\tilde{H},E\right)$ over *X* which is described in Example 7 is a closed hesitant fuzzy soft ideal over *X*.

**Theorem 23 .**

In a *BCI*-algebra *X*, every closed hesitant fuzzy soft ideal over *X* based on a parameter is a hesitant fuzzy soft subalgerba over *X* based on the same parameter.

**Proof**

Let $\left(\tilde{H},A\right)$ be a closed hesitant fuzzy soft ideal over *X* based on a parameter *e* ∈ *A*. Then $h_{\tilde{H}[e]}(x)\subseteq h_{\tilde{H}[e]}(0*x)$ for all *x* ∈ *X*. It follows that

(33) hH~[e](x∗y)⊇hH~[e]((x∗y)∗x)∩hH~[e](x)=hH~[e](0∗y)∩hH~[e](x)⊇hH~[e](x)∩hH~[e](y),

for all *x*, *y* ∈ *X*. Therefore $\left(\tilde{H},A\right)$ is a hesitant fuzzy soft subalgebra over *X* based on the parameter *e* ∈ *A*.

**Theorem 24 .**

Let $\left(\tilde{H},A\right)$ be a hesitant fuzzy soft ideal over a *BCI*-algebra *X* based on a parameter *e* ∈ *A*. Then it is closed if and only if it satisfies

(34) $$\begin{array}{c} \left(\forall x,y\in X\right)\;\left(h_{\tilde{H}[e]}\left(x*y\right)⊇h_{\tilde{H}[e]}\left(x\right)\cap h_{\tilde{H}[e]}\left(y\right)\right). \end{array}$$

**Proof**

Assume that $\left(\tilde{H},A\right)$ is a closed hesitant fuzzy soft ideal over a* BCI*-algebra *X* based on a parameter *e* ∈ *A*. Since (*x*∗*y*)∗*x* ≤ 0∗*y* for all *x*, *y* ∈ *X*, it follows from (21) that

(35) hH~[e](x∗y)⊇hH~[e](x)∩hH~[e](0∗y)⊇hH~[e](x)∩hH~[e](y),

for all *x*, *y* ∈ *X*.

Conversely, let $\left(\tilde{H},A\right)$ be a hesitant fuzzy soft ideal over a* BCI*-algebra *X* based on a parameter *e* ∈ *A* that satisfies the condition (34). Since $h_{\tilde{H}[e]}(x)\subseteq h_{\tilde{H}[e]}(0)$ for all *x* ∈ *X*, we have

(36) $$\begin{array}{c} h_{\tilde{H}[e]}\left(0*x\right)⊇h_{\tilde{H}[e]}\left(0\right)\cap h_{\tilde{H}[e]}\left(x\right)=h_{\tilde{H}[e]}\left(x\right), \end{array}$$

for all *x* ∈ *X*. Therefore $\left(\tilde{H},A\right)$ is a closed hesitant fuzzy soft ideal over a* BCI*-algebra *X* based on a parameter *e* ∈ *A*.

## 5. Conclusion

As another generalization of the standard soft sets, the concept of hesitant fuzzy soft sets has been introduced in [6, 16]. They have presented an application of hesitant fuzzy soft soft sets in a decision making problem. The first author [20] has applied the notion of soft sets by Molodtsov to the theory of* BCK*/*BCI*-algebras. Also, Jun et al. [21] have discussed soft set theory which is applied to ideals in *d*-algebras. In this paper, we applied the notion of hesitant fuzzy soft sets to the theory of* BCK/BCI*-algebras. We introduced the concepts of hesitant fuzzy soft subalgebras and (closed) hesitant fuzzy soft ideals, and then we investigated related properties. We provided relations between a hesitant fuzzy soft subalgebra and a hesitant fuzzy soft ideal. We gave conditions for a hesitant fuzzy soft set to be a hesitant fuzzy soft subalgebra. We also provided conditions for a hesitant fuzzy soft subalgebra to be a hesitant fuzzy soft ideal. We discussed characterizations of a (closed) hesitant fuzzy soft ideal. On the basis of these results, we will study applications of hesitant fuzzy soft sets to several ideals and filters of* BCK/BCI*-algebras,* MTL*-algebras, *MV*-algebras, *R*_0_-algebras, and so forth.

## Figures and Tables

**Table 1:** Tabular representation of the hesitant fuzzy soft set $\left(\tilde{H},A\right)$.

$\tilde{H}$	0	*a*	*b*	*c*	*d*
*e*_1_	[0.2,0.8]	[0.2,0.8]	[0.2,0.8]	(0.2,0.3)	(0.2,0.3)
*e*_2_	[0.1,0.9]	[0.1,0.2)∪(0.2,0.8]	{0.3}	[0.3,0.6]	{0.3}
*e*_3_	[0.3,0.7]	{0.4}	[0.3,0.5]	{0.4}	{0.4}

**Table 2:** Tabular representation of the hesitant fuzzy soft set $\left(\tilde{G},B\right)$.

$\tilde{G}$	0	*a*	*b*	*c*	*d*
*e*_3_	[0.3,0.7]	[0.3,0.7]	[0.3,0.7]	{0.3,0.7}	{0.3,0.7}
*e*_4_	{0.3}	[0.2,0.6)	[0.3,0.4]	[0.3,0.4]	{0.3,0.4}
*e*_5_	[0.2,0.5]	(0.2,0.3)	[0.2,0.5)	(0.2,0.3)	(0.2,0.3)

**Table 3:** Tabular representation of the hesitant fuzzy soft set $\left(\tilde{H},E\right)$.

$\tilde{H}$	0	1	2	*a*	*b*
*e*_1_	[0.2,0.8]	[0.2,0.5]	[0.2,0.3]	{0.2}	{0.2}
*e*_2_	[0.3,0.5)	{0.3}	[0.3,0.5)	[0.3,0.4]	{0.3}
*e*_3_	[0.2,0.6]	{0.2}	[0.2,0.4]	[0.2,0.3]	{0.2}

**Table 4:** Tabular representation of the hesitant fuzzy soft set $\left(\tilde{H},A\right)$.

$\tilde{H}$	0	*a*	*b*	*c*	*d*
*e*_1_	[0.3,0.8]	[0.3,0.8]	[0.3,0.8]	[0.3,0.5]	[0.3,0.5]
*e*_2_	[0.3,0.9)	[0.3,0.8)	{0.3}	[0.3,0.6]	{0.3}
*e*_3_	[0.1,0.7]	{0.1}	[0.1,0.5]	{0.1}	{0.1}
*e*_4_	{0.1}	[0.1,0.2]	[0.1,0.3]	[0.1,0.4]	[0.1,0.5)
*e*_5_	[0.1,0.2]	{0.1}	[0.1,0.5]	[0.1,0.6)	{0.1}

**Table 5:** Tabular representation of the hesitant fuzzy soft set $\left(\tilde{H},E\right)$.

$\tilde{H}$	Apple	Banana	Carrot	Melon	Peach
Cat	[0.2,0.5]	[0.2,0.3]	[0.2,0.3]	[0.2,0.3]	[0.2,0.3]
Cow	[0.3,0.6)	[0.3,0.4)	[0.3,0.6)	[0.3,0.4)	[0.3,0.4)
Dog	[0.2,0.7]	[0.2,0.7]	{0.2}	[0.2,0.7]	{0.2}
Duck	[0.1,0.3]	{0.1}	[0.1,0.3]	{0.1}	{0.1}
Horse	[0.3,0.4)	[0.3,0.4)	[0.3,0.9)	{0.3}	[0.3,0.6)
Pig	[0.1,0.5)	[0.1,0.4]	{0.1}	[0.1,0.4]	{0.1}

**Table 6:** Tabular representation of the hesitant fuzzy soft set $\left(\tilde{H},B\right)$.

$\tilde{H}$	Apple	Banana	Grape	Melon	Peach
Duck	{0.3}	{0.3}	[0.3,0.7]	{0.3}	[0.3,0.5)
Horse	[0.2,0.5)	{0.2}	[0.2,0.6)	{0.2}	{0.2}
Pig	[0.3,0.4)	[0.3,0.4]	[0.3,0.8)	[0.3,0.6]	[0.3,0.7)
